# The complete chloroplast genome sequence of *Bolboschoenus planiculmis* (Cyperaceae)

**DOI:** 10.1080/23802359.2021.2024773

**Published:** 2022-01-24

**Authors:** Yu Ning, Shu Bin Dong, Yu Xin Zhao, Zhe Pan, Hua Ma

**Affiliations:** a Institute of Wetland Research, Chinese Academy of Forestry Research, Beijing, China; b College of Biological Sciences and Technology, Beijing Forestry University, Beijing, China; c Research Center of Ecological Civilization, Sichuan Academy of Environmental Policy and Planning, Chengdu, Sichuan, China; d Zoige Alpine Wetland Ecosystem Research Station, Zoige, Sichuan, China

**Keywords:** *Bolboschoenus planiculmis*, Cyperaceae, complete chloroplast genome

## Abstract

*Bolboschoenus planiculmis* is a typical wetland sedge with both ecological and agricultural value. We report the first complete chloroplast genome sequence of this species. The total genome size is 186,539 bp, containing a large single-copy region (LSC) of 104,654 bp, a small single copy region (SSC) of 9,659 bp and two inverted repeats (IRs) of 36,113 bp by each. The GC content is 33.59%. The genome encodes 105 unique genes, including 71 protein-coding genes, 30 tRNA genes, and 4 rRNA genes. Phylogenetic analysis shows this species has a strong sister relationship with *Cyperus*. Our work could be helpful in understanding the evolution of Cyperaceae.

*Bolboschoenus planiculmis* (F. Schmidt) T. V. Egorova is a typical sedge in wetland habitats. Naturally, *B. planiculmis* often tends to reproduce clonally, which enables it to survive adverse conditions and expand quickly when opportunity windows come (Herben and Klimešová 2020; Stuefer et al. 1994). This biological feature makes *B. planiculmis* a malignant weed when it thrives in paddy fields (Im et al. 2003). Nevertheless, due to the high starch content of its corm, this plant can support many wetland birds when their food sources are insufficient. Some literature even shows its dietary use in human society with ethnical features rooted in ancient times (Heiser 1978; Amat 1995). Recent studies also confirm its performance in water decontamination (Madejón et al. 2006; Santos et al. 2015). By far, molecular information is extremely scarce for genus *Bolboschoenus*. Here we report the first complete chloroplast genome of *B. planiculmis*, which will be helpful in future work on Cyperaceae.

The samples were collected at Chang Gou wetland, Beijing, China (GPS: E115°53′24″, N 39°35′56.4″). Fresh leaves were collected and wrapped in Drikold, then transferred to be stored at −80 °C lab environment. The relevant specimen was deposited at the herbarium of Beijing Museum of Nature History (BJM0272524, contact xiaxiaofei@bmnh.org.cn). The total genomic DNA was extracted using DP305-3 plant genomic DNA Kit (Tiangen, Beijing, China) according to the standard protocol. The sequencing platform was Illumina NovaSeq 6000. Totally, *ca* 21.4 million high-quality clean reads (PE 150) were generated. Aligning, assembly and annotation were conducted by bowtie2(v2.2.4) (Langmead and Salzberg 2012), SPAdes(v3.10.1) (Bankevich et al. 2012), SSPACE(v2.0) (Boetzer et al. 2011), Gapfiller(v2.1.1) (Nadalin et al. 2012). The assembled and annotated chloroplast genome sequence has been submitted to Genbank database under the accession number MZ751078.1.

The plastome of *B. planiculmis* is a circular DNA molecule with a length of 186,539 bp, a comparable genome size to several published chloroplast genome. The full plastome consists of a large single-copy region (LSC) of 104,654 bp, small copy region (SSC) of 9,659 bp and 2 inverted repeats (IRs) of 36,113 bp by each. The overall GC content of *B. planiculmis* chloroplast genome was 33.59%, with corresponding GC values of the LSC, SSC and IR regions for 31.38, 26.76, and 37.68%, respectively. The chloroplast genome of *B. planiculmis* comprises 136 genes, among which, 105 are unique genes, including 71 protein-coding genes, 4 ribosomal RNA (rRNA) genes, and 30 transfer RNA (tRNA) genes.

A maximum likelihood tree has been built to demonstrate the evolutionary positon of *B. planiculmis*. Complete chloroplast genomes of 12 species have been used to construct the phylogeny, including 9 published chloroplast genomes of Cyperaceae and two accessions of Poaceae from NCBI (*Oryza sativa* and *Aegilops tauschii* as the outgroup). The sequences were aligned using the default settings in MAFFT v7 (Katoh et al. 2019). The phylogenetic analyses were conducted using IQTREE v1.6.7 (Nguyen et al. 2015). The fitted model was TVM + F+G4, with branch support tested by SH-aLRT (1000 replicates) and ultrafast bootstrap (1000 replicates). According to the current sampling extent, our result presents a phylogeny with most internal relationships well supported. The sisterhood of *Bolboschoenus planiculmis* with genus *Cyperus* has been confirmed. This research could be helpful to further researches in Cyperacea phylogeny and the practical value of this promising plant.

## Data Availability

The data that support the findings of this study are openly available in the Genbank database at https://www.ncbi.nlm.nih.gov/, under accession number [MZ751078.1]. The associated BioProject, SRA, and BioSample numbers are PRJNA771475, SRR16350061, and SAMN22310869 respectively. Phylogenetic relationships among 12 complete chloroplast genomes. Branch scores are given as (SH-aLRT value/ultrafast bootstrap value). The analyzed species are shown with the corresponding Genbank accession numbers in parentheses.
